# Association between pediatric Risk, Injury, Failure, Loss and End Stage Renal Disease score and mortality in a pediatric intensive care unit: a retrospective study

**DOI:** 10.5935/0103-507X.20180065

**Published:** 2018

**Authors:** Jader Pereira Almeida, Ivan Ferraz Valente, Marina da Rocha Lordelo

**Affiliations:** 1 Unidade de Terapia Intensiva, Hospital Pequeno Príncipe - Curitiba (PR), Brasil.; 2 Unidade de Terapia Intensiva, Hospital Martagão Gesteira - Salvador (BA), Brasil.; 3 Serviço de Nefrologia Pediátrica, Hospital Martagão Gesteira - Salvador (BA), Brasil.

**Keywords:** Acute kidney injury, Mortality, Intensive care units, pediatric

## Abstract

**Objective:**

To evaluate the association between acute kidney injury through the pediatric Risk, Injury, Failure, Loss and End Stage Renal Disease score and mortality in a pediatric intensive care unit.

**Methods:**

This retrospective cohort study assessed all children admitted to the pediatric intensive care unit of a reference hospital in Brazil from January to December 2016. Patients were screened for the presence of acute kidney injury through the pediatric Risk, Injury, Failure, Loss and End Stage Renal Disease score. Patients were subdivided into the stages of Risk, Injury and Kidney Failure.

**Results:**

The sample comprised 192 children, of whom 45.8% developed acute kidney injury, with 79.5% of the cases identified up to 72 hours after admission. Patients with acute kidney injury showed a 3.74 increase risk of death (p = 0.01) than the control group. Patients with kidney failure had a mortality rate that was 8.56 times greater than that of the remaining sample (p < 0.001). The variables that were associated with the stages of acute kidney injury were nephrotoxic drugs (p = 0.025), renal replacement therapy (p < 0.001), vasoactive drugs (p < 0.001), pediatric risk of mortality 2 score (p = 0.023), fluid overload (p = 0.005), pediatric intensive care unit length of stay (p = 0.001) and death (p < 0.001).

**Conclusion:**

In this study, the pediatric Risk, Injury, Failure, Loss and End Stage Renal Disease score proved to be a useful tool for the early identification of severely ill children with acute kidney injury, showing an association with mortality. We thus suggest its use for pediatric intensive care unit patient admission.

## INTRODUCTION

Acute kidney injury (AKI) is a complication that frequently occurs in pediatric patients in a severe state. It is characterized by an instability of the body homeostasis and, consequently, hemodynamic impairment.^(1)^ Its incidence has been associated with an increased mortality rate, an extended time in pediatric intensive care units (PICU) and increased admission costs.^(1-3)^

The most common marker used in clinical practice to identify AKI is the serum creatinine level.^(4,5)^ Despite its widespread dissemination, researchers have indicated that slight elevations of serum creatinine may result in severe outcomes, which suggests it is not efficient as an early marker, but rather a late marker for kidney dysfunction.^(6)^ The discovery of new markers, such as Neutrophil Gelatinase Associated Lipocalin, Kidney Injury Molecule-1 and Interleukin-18, have broadened the horizons in the identification of AKI in its early stage. However, these exams have not been disseminated in clinical practice due to their low availability and high cost.^(7-9)^

Given the occurrence of a lack of solid criteria for AKI definition, the Acute Dialysis Quality Initiative group, formed by specialists in nephrology and intensive care, created a score referred to as Risk, Injury, Failure, Loss and End Stage Renal Disease (RIFLE)^(10)^ in 2004. Akcan-Arikan et al. subsequently adapted this model to pediatric patients in 2007. It was named pediatric RIFLE (pRIFLE), which classifies the degree of kidney dysfunction by the patient’s clearance of creatinine (ClCr) and urinary output in relation to their weight.^(11)^

Despite the advances on the subject, scientific studies have indicated imparities regarding the prognosis of AKI patients. Akcan-Arikan et al. did not identify a statistically significant result regarding mortality between groups with and without AKI in their prospective study that analyzed 150 severe patients, despite the observation that patients with kidney failure had a mortality rate that was 2.36 times greater than that of the remaining cohort (25.8% *versus* 10.9%, respectively, p = 0.03).^(11)^ However, Cabral et al. not only managed to identify a significant association between AKI and death (p = 0.02) but also a difference in the mortality rate that was 15 times greater in kidney failure patients than in the remaining sample (53.8% *versus* 3.5%, respectively, p < 0.001).^(12)^

In this sense, this paper intends to evaluate the association of the pRIFLE score with the mortality of admitted children in the PICU of a reference hospital in Brazil.

## METHODS

This paper is a retrospective cohort study performed with all admitted children in the PICU of a referral tertiary pediatric hospital, from January to December of 2016. The institution has a capacity for 10 PICU beds. The data were extracted from the electronic medical records of the unit. The criteria used to include patients in the research were a minimum of a 24 hour stay in the PICU and an age between 28 days and 14 years. The exclusion criteria included patients with chronic nephropathy, defined by a glomerular filtration rate < 35mL/min/1.73m^2^ for more than 4 weeks;^(11)^ patients who were postoperative following cardiac surgery; and patients who presented the loss of necessary anthropometric data to calculate the variables of interest. This study was approved by a Research Ethics Committee, respecting Resolution 466/12.

The variables of interest included sex, age, reason for admission, use of vasoactive drugs (VAD), duration of mechanical ventilation (MV), use of nephrotoxic drugs, fluid overload, renal replacement therapy (RRT), PICU length of stay, hospital length of stay and death. Moreover, the gravity scores of the Pediatric Index of Mortality 2 (PIM2) and the Pediatric Risk of Mortality 2 (PRISM2) were evaluated.

All patients were screened and assessed regarding the presence of AKI during their stay in the PICU through the pRIFLE score for a maximum period of 14 days since admission. Children with an estimated CrCL (eCrCl) within 25% of normal were classified as not having AKI; children with an eCrCl decreased by 25 - 50% were classified as pRIFLE Risk; children with an eCrCl decreased by 50 - 75% were classified as pRIFLE Injury; and children with an eCrCl decreased by 75% or more were classified as pRIFLE Failure.^(11)^ The calculation of the baseline eCrCl was performed through the creatinine serum level, using enzymatic method, from the last 3 months prior to admission to the PICU through the revised Schwartz formula.^(13)^ The baseline eCrCl of 100mL/min/1.73m^2^ was used as proposed by Akcan-Arikan et al.,^(11)^ when it was not possible to calculate. The calculation of fluid overload was performed as indicated in Goldstein et al.^(14)^

Epi Info^TM^ Windows software 7.2 version was used to perform the statistical analyses. Calculations of the frequencies and proportions of the qualitative variables were performed. For the quantitative variables, the mean, the standard deviation (SD), the median, and the maximum and minimum variations were calculated. The associations of the variables were determined using Chi-square and Fischer’s Exact tests, as well as logistic regression for odds ratio (OR) calculation and the adjustment of potential confounders, with a 95% confidence interval (95%CI) and significance level of 5%.

## RESULTS

Four hundred twelve patients were admitted to the PICU in a period of 12 months. Of these patients, 220 patients were excluded, resulting in 192 patients. Of the excluded patients, 118 patients were postoperative following cardiac surgery; 45 patients had a time of admission shorter than 24 hours; 34 patients lacked the necessary anthropometric data for the calculation of the variables of interest; 12 patients exhibited chronic nephropathy; 9 patients were older than 14 years; and 2 patients were transferred to other health clinics for logistical reasons.

In summary, 60.4% (116) of the patients were male, with a mean age of 4.3 (median: 2 years, SD ± 4.2 years, minimum: 1 month and maximum: 14 years). Mechanic ventilation was used in 39% (75) of the sample, with a mean 5.4 days of use (median: 4 days, standard deviation ± 5, minimum: 0 days and maximum: 20 days), 26% (50) of the sample used VAD, 8.3% (16) of the patients used RRT and 9.9% (19) of the patients died (Table 1).

**Table 1:** Clinical-demographic profile of the studied sample

Variables	Value
Male sex	60.4
Age (years)	2 ± 4.2
Oncological patients	25
Baseline eClCr (mL/1,73m^2^/min)	163.7 ± 92.4
Use of VAD	26
MV (days)	5,4 ± 5
When AKI was developed (days)	2 ± 2.3
AKI	45.8
AKI stages	
Risk	46.6
Injury	28.4
Failure	25
RRT	8.3
PIM2	10 ± 22.1
PRISM2	3.55 ± 7.23
Hospital LOS	29.4 ± 33.6
PICU LOS	7 ± 14.9
Death	9.9

eClCr - estimated clearance of creatinine; VAD - vasoactive drugs; MV - mechanical ventilation; AKI - acute kidney injury; RRT - renal replacement therapy; PIM2 - Pediatric Index of Mortality 2; PRISM2 - Pediatric Risk of Mortality 2; PICU - pediatric intensive care unit; LOS - length of stay. Values are expressed as percentage or mean ± standard deviation.

Regarding the gravity scores used in the PICU admission, the PIM2 had a mean death expectancy of 10% (median: 1.7%, SD ± 22.1, varying between 0.8% and 100%), while the PRISM2 had a mean of 3.55% (median: 1.3%, SD ± 7.23, varying between 0% and 64.6%).

In a decreasing order of frequency, 28.6% (55) of the sample was postoperative following pediatric surgery, 24% (46) had respiratory failure, 22.9% (44) were admitted for sepsis/septic shock, 14.1% (27) were postoperative following neurosurgery and 10.4% (20) had other pathologies that included cardiac, neurological, endocrine and hematological diseases.

In relation to the AKI classification through the pRIFLE score, 45.8% (88) of the children developed AKI at some point during the PICU admission wherein the maximum pRIFLE of 79.5% (70) of the sample was identified in the first 72 hours of admission. When subclassified, 46.6% (41/88) had a pRIFLE “risk”; 28.4% (25/88) had pRIFLE “injury”; and 25% (22/88) had pRIFLE “failure”. The pRIFLE was calculated through the eClCr alone in 76% (67) of the patients, only via the urinary output in 9.1% (8) of the patients, and by both methods in 14.9% of the patients.^(13)^^)^ The baseline eCrCl was identified in 47.9% (92) of the sample and had a mean of 163mL/min/1.73m^2^ (median: 142mL/min/1.73m^2^, SD ± 92.4, varying between 55 and 779mL/min/1.73m^2^).

Regarding the use of nephrotoxic drugs, 14% (27) of the patients used 2 or more drugs; 25% (48) used 1 drug; and 61% (117) did not use any drugs. Iodinated contrast was the most used nephrotoxic drug and was present in 17.2% of the sample, followed by vancomycin 15.1%; nonsteroidal anti-inflammatory drugs 10.9%; aminoglycosides 10.4%; amphotericin B 8.3%; and chemotherapy for the treatment of neoplasia 6.3%.

Of the patients who died (19), 73.7% (14) had AKI. Of these patients, 7.2% (1) developed a pRIFLE “risk”; 21.4% (3) had pRIFLE “injury”; and 71.4% (10) had pRIFLE “failure”. The patients with AKI had an increased risk of death compared to patients without AKI (OR: 3.74; 95%CI: 1.29 - 10.86). When we evaluated the association between AKI and mortality through multivariate logistic regression adjusted for age, fluid overload and PRISM2, there was a loss of statistical significance (OR: 1.6; 95%CI: 0.43 - 5.91). However, when the same analysis was performed separating the case group in patients with “injury” and “renal failure” and the patients with pRIFLE “risk” were included in the group without AKI, a statistically significant association was identified when adjusted for the same variables (OR: 5.2, 95%CI: 1.42 - 19.01) (Table 2). The patients with Kidney Failure had a mortality rate that was 8.56 times greater than that of the remaining sample (45.4% *versus* 5.3%, respectively; p < 0,001).

**Table 2:** Multivariate logistic regression for the adjustment of potential confounding factors in the association between acute kidney injury and mortality

AKI	Mortality OR (95%CI)
Risk + injury + failure (reference group: without AKI)	
Unadjusted	3.74 (1.29 - 10.86)
Adjusted - age	3.66 (1.25 -10.74)
Adjusted - fluid overload*	2.52 (0.82 - 7.71)
Adjusted - PRISM2†	1.67 (0.43 - 5.91)
Adjusted - age, fluid overload and PRISM2	1.60 (0.43 - 5.91)
Injury + failure (reference group: risk + without AKI)	
Unadjusted	10.63 (3.55 - 31.85)
Adjusted - age	11.36 (3.71 - 34.8)
Adjusted - fluid overload*	6.93 (2.15 - 22.3)
Adjusted - PRISM2†	5.79 (1.76 - 19.01)
Adjusted - age, fluid overload and PRISM2	5.2 (1.42 -19.01)

AKI - acute kidney injury; OR - odds ratio; 95%CI - 95% confidence interval; PRISM2 - Pediatric Risk of Mortality 2.

* Fluid overload ≥ 10%;

† PRISM2 ≥ 10%.

The mean duration of PICU admission was 7 days (median: 3 days, SD ± 14.9, varying between 1 and 162 days), and the mean duration of hospitalization was 29.4 days (median: 17.5 days, SD: ± 33.6, varying between 2 and 214 days). The patients with AKI had an association with a longer PICU length of stay than the patients without AKI (OR: 2.78; 95%CI: 1.41 - 5.49); however, a significant relationship was not found with a longer hospitalization (OR: 1.73, 95%CI: 0.97 - 3.09).

The variables that were associated with the stages of AKI included the use of nephrotoxic drugs (p = 0.025), RRT (p < 0.001), VAD (p < 0.001), PRISM2 (p = 0.023), fluid overload (p = 0.005), PICU length of stay (p < 0.001) and death (p < 0.001). The variables sex, duration of MV, PIM2 and hospital length of stay did not present a statistically meaningful association (Tables 3 and 4). The age variable also did not present a statistically significant relationship with the AKI stages, although it was observed that infants had an increased risk of AKI [risk + injury + failure *versus* no-AKI] compared to adolescents (OR: 2.65; 95%CI: 1.12 - 6.25).

**Table 3:** Association of clinical-demographic variables with the pediatric Risk, Injury, Failure, Loss and Stage Renal Disease Score stages

AKI stage	Risk N (%)	Injury N (%)	Failure N (%)	Total	p value
Sex					SI
Male	25 (43.1)	17 (29.3)	16 (27.6)	58	
Female	16 (53.3)	8 (26.7)	6 (20)	30	
Age					SI
Infant	20 (47.6)	9 (21.4)	13 (31)	42	
Preschool	8 (40)	8 40.0)	4 (20)	20	
School	7 (43.7)	6 (37.5)	3 (18.8)	16	
Teenager	6 (60)	2 (20)	2 (20)	10	
Nephrotoxic drugs					0.025
None	23 (53.5)	12 (27.9)	8 (18.6)	43	
1	13 (56.5)	7 (30.5)	3 (13)	23	
≥ 2	5 (22.7)	6 (27.3)	11 (50)	22	
PIM2					SI
< 10	32 (54.2)	16 (27.1)	11 (18.7)	59	
≥ 10	9 (31.0)	9 (31)	11 (38)	29	
PRISM2					0.023
< 10	40 (50)	23 (28.8)	17 (21.2)	80	
≥ 10	1 (12.5)	2 (25)	5 (62.5)	8	
Total	41	25	22	88	

AKI - acute kidney injury; PIM2 - Pediatric Index of Mortality 2; PRISM2 - Pediatric Risk of Mortality 2; SI - statistically insignificant.

**Table 4:** Association of the pediatric Risk, Injury, Failure, Loss and Stage Renal Disease Score stages with the clinical outcomes of the studied sample

AKI stage	Risk N (%)	Injury N (%)	Failure N (%)	Total	p value
RRT					< 0.001
Yes	-	4 (16)	12 (54.5)	16	
No	41 (100)	21 (84)	10 (45.5)	72	
MV (days)					SI
< 7	9 (75)	8 (53.3)	8 (52.4)	25	
≥ 7	3 (25)	7 (46.7)	10 (47.6)	20	
VAD					< 0.001
Yes	3 (7.3)	12 (48)	22 (100)	37	
No	38 (92.7)	13 (52)	-	51	
Fluid overload					0.005
< 10	32 (78)	13 (52)	8 (36.4)	53	
10 - 20	6 (14.6)	9 (36)	6 (27.2)	21	
> 20	3 (7.4)	3 (12)	8 (36.4)	14	
Hospital LOS (days)					SI
< 20	23 (56.1)	13 (52)	8 (36.4)	44	
≥ 20	18 (43.9)	12 (48)	14 (63.6)	44	
PICU LOS (days)					< 0.001
< 7	32 (78.0)	18 (72.0)	7 (31.8)	57	
≥ 7	9 (22.0)	7 (28.0)	15 (68.2)	31	
Death					< 0.001
Yes	1 (2.4)	3 (12.0)	10 (45.5)	14	
No	40 (97.6)	22 (88.0)	12 (54.5)	74	

AKI - acute kidney injury; RRT - renal replacement therapy; MV - mechanical ventilation; VAD - vasoactive drugs; LOS - length of stay; PICU - pediatric intensive care unit; SI - statistically insignificant.

## DISCUSSION

Patients with kidney dysfunction present an impairment of the primordial functions for the proper functioning of the body, such as a hydroelectrolyte imbalance and acid-base disorders, in addition to an accumulation of toxic substances in the body. These impairments may result in an increase in the mortality rate.

In this study, patients who developed AKI, classified by the pRIFLE score, were 3.74 times more likely to die than patients without AKI (p = 0.014). This result is similar to those of Cabral et al.^(12)^ and Bresolin et al.,^(15)^ who compared the two groups and identified an increase in the mortality rate of 2.53 and 5 times, respectively. Moreover, the progressive increase in the number of deaths increased with more serious AKI stages, particularly the “failure“criteria, as observed in other studies.^(11,12,15,16)^

Furthermore, when groups were redistributed among “injury + failure” and “no-AKI + risk”, there was a statistically significant relationship between AKI and mortality, even when confounding factors, such as age, PRISM2 and fluid overload, were adjusted through multivariate logistic regression, in contrast to other studies.^(1,16)^

Several hypotheses may be proposed. First, the different clinical and demographical profiles of the studied populations may influence the final results. Another factor that could justify the divergence of the results is that the majority of the studies did not use a uniform methodology concerning the use of the pRIFLE score. Some studies only used the eClCr as a qualifying criterion and did not employ urinary output as suggested by Akcan-Akiran et al.^(11,12,16,17)^ Moreover, a low availability of basal Creatinine was identified in most studies, which is necessary for the eCrCl calculation. While the original paper^(11)^ used the baseline eClCr of 73% of the sample, Hui et al.^(18)^ identified it in 46% of the patients. In this sample, it was possible to observe these data in 47.9% of the children.

These observations are important due to the assumption that the use of the eClCr of 100mL/min/1.73m^2^, as proposed by Akcan-Arikan et al.,^(11)^ in the absence of a baseline eClCr, may underestimate the incidence of AKI because the standard baseline eClCr in the studies varies between 100 to 197mL/min/1,73m^2^.^(15,18,19)^ Moreover, some authors have opted to use the baseline eClCr in 120mL/min/1,73m^2^.^(1,12,16,20)^ These data suggest that the incidence of AKI may be different depending on how the pRIFLE qualifying criteria are employed.

In addition to mortality, other variables also present an association with AKI in different stages. In our research, nephrotoxic drugs, VAD and RRT had greater associations with pRIFLE “injury and failure”. The classification of AKI by the pRIFLE was also related to the fluid overload and PICU length of stay, and these data are similar to those found in the literature.^(1,12,15,19-22)^ However, our study did not identify an association with this score and hospital length of stay (p = 0.319), which is different from the previous studies of Soler et al.,^(1)^ Akcan-Arikan et al.,^(11)^ Bresolin et at.,^(15)^ Naik et al.^(16)^ and Palmieri et al.^(19)^ Social and logistical issues may be the reasons for these data, as our study was conducted in a philanthropic institution that treats a low-income population, with social pendencies before their discharge from the hospital.

Comparing the pRIFLE with the mortality scores that are commonly used in PICU patient admission, our results did not show an association between the different stages of AKI and PIM2 (p = 0.073), although an association with PRISM2 (p = 0.023) was identified. Cabral et al.^(12)^ showed that the PIM2 may underestimate the mortality rate of pediatric patients with severe AKI, probably because its classification does not involve renal function as a prognostic criterion. However, the PRISM2 presents divergent results in the research literature; thus, additional research is necessary to evaluate its association with AKI.^(1,19)^

When the pRIFLE is used in PICU patient admission, studies show that AKI occurs early. In the first 24 hours, studies show that it is possible to identify approximately 33 - 81.5% of patients who contracted AKI at some point of the PICU admission.^(11,15,20,21)^ During the first 72 hours, this number increases to 50 - 99% of the cases.^(1,12,16,22)^ These results are similar to our study, which could identify 79.5% of the AKI patients in the same period. Thus, the pRIFLE may be a useful tool for the early identification of severe AKI patients.

Our study presented several limitations. It was determined that 8.25% of the children were excluded due to the loss of anthropometrics. Although the size of the sample is compatible with those of other samples, this issue may influence the results obtained. The data were collected in one unit only, with the intrinsic limitations of a retrospective study. The first author was responsible for the research data collection, which was minimized by the standardization in the completion of a structured questionnaire. In relation to the nephrotoxic drugs, medication groups were used in the form different classes, such as cancer chemotherapeutics, aminoglycosides and non-steroidal anti-inflammatory drugs. Finally, this paper focused only on the maximum pRIFLE and did not detail the AKI evolution during the PICU admission.

## CONCLUSION

In this paper, the pediatric Risk, Injury, Failure, Loss and End Stage Renal Disease score proved to be a useful tool for the early identification of severe acute kidney injury children. This score shows an association with mortality, particularly when it meets the pRIFLE injury or failure criteria. Thus, we suggest its use for pediatric intensive care unit patient admission.

Furthermore, it is noted that the way patients are selected in research and how the pediatric Risk, Injury, Failure, Loss and End Stage Renal Disease qualifying criteria are employed may impact the incidence of acute kidney injury. Consequently, it can also impact the incidence of mortality, which indicates a greater standardization is necessary for studies related to this issue.
